# Investigation of the Tensile Strength of Adhesive-Bonded Steels Using Surface Treatments

**DOI:** 10.3390/ma16247663

**Published:** 2023-12-15

**Authors:** Péter Kovács, Benjámin Körömi, Zoltán Weltsch, Miklós Berczeli

**Affiliations:** 1 Doctoral School of Multidisciplinary Engineering Sciences, Szechenyi Istvan University, H-9000 Gyor, Hungary; kovacs.peter2@nje.hu; 2 Department of Innovative Vehicles and Materials, GAMF Faculty of Engineering and Computer Science, John von Neumann University, H-6000 Kecskemet, Hungary; benjaminkoromi@gmail.com; 3 Department of Automotive and Railway Engineering, ZalaZONE Innovation Park, Széchenyi István University, H-9000 Gyor, Hungary; weltsch.zoltan@sze.hu

**Keywords:** adhesive, steel, surface treatment, plasma, wetting, tensile strength

## Abstract

This study explores the tensile strength of adhesive joints in steel, focusing on the influence of heat treatment and diverse surface modifications. Results indicate a notable relationship between annealing temperature and tensile strength, with the most favorable outcomes identified at 90 min and 165 °C. Particularly, surfaces treated through turning, sandblasting, and plasma treatment (type C) consistently outperformed other methods. A standout revelation emerged from the turned, sandblasted, and plasma-treated surface (C), showcasing an exceptional tensile strength of 69.06 MPa. Load-holding tests underscored its resilience under diverse load conditions. Surface analyses, including roughness measurements, wetting characteristics, and Scanning Electron Microscope imaging, provided valuable insights into structural transformations induced by different treatments. Chemical composition examinations unveiled significant alterations post-plasma treatment, impacting surface chemistry and contributing to an outstanding tensile strength of 67.63 MPa. In essence, this research offers a glimpse into the nuanced factors influencing adhesive joint strength in steel. The turned, sandblasted, and plasma-treated surface emerges as a promising avenue, sparking further curiosity into the underlying mechanisms propelling superior tensile strength in adhesive joints.

## 1. Introduction

The adhesion bond is created by intermolecular forces. These forces come from second-order chemical bonds, which can be van der Waals forces or hydrogen bonds. Van der Waals forces can be composed of three parts: interactions from a constant dipole (orientation interaction), interactions from an induced dipole (induction interaction), and dispersion forces (dispersion interaction) [1]. These interactions depend on the chemical structure and the distance between molecules. An orientation interaction is created between two molecules with constant dipole moments by the attraction of dipoles that are properly oriented with respect to each other [2]. The magnitude of the interaction is directly proportional to the magnitude of the dipole moments. Increasing the temperature increases the thermal motion, so the energy of the dipole–dipole interaction decreases with increasing temperature. Induction interactions occur when molecules with a constant dipole moment induce electron shifts on a neighboring polarizable apolar molecule, i.e., an induced dipole [3]. The magnitude of the induction interaction depends on the dipole moment and the polarizability and is independent of temperature over a wide temperature range. Dispersion interactions are the result of the electron cloud of molecules in motion. As a result, dipoles are formed that vary in time and space and interact with each other. The magnitude of the dispersion interaction depends on the degree of charge fluctuations and the polarizability of the molecules. This interaction occurs in all molecules and is a fundamental form of interaction between apolar molecules. A special form of interaction between molecules is hydrogen bonding (hydrogen bonding) [4]. This occurs when the hydrogen atom in a covalent bond is attached to an atom with high electronegativity (fluorine, oxygen, nitrogen) [5]. In this case, the bond is very strongly polarized, i.e., there is a near unity of charge at the ends of the dipoles. This is compounded by the fact that these atoms are very small, so the charge is concentrated in a small space [6]. This causes a very large interaction between the dipoles, which is called the hydrogen bond structure. The energy of hydrogen bonding is significantly higher than van der Waals interactions and can lead to the formation of molecular associates [7].

There are several theories about adhesion [8]. One such theory is the theory of adsorption [5]. According to adsorption theory, adhesion is the result of molecular contact between two substances and surface forces [9]. Adhesion involves the adsorption of adhesive molecules on the substrate [10] and the resulting attractive forces [11], usually called secondary or van der Waals forces [12]. For these forces to develop, the corresponding surfaces should not be separated by more than five angstroms. Therefore, the adhesive must come into molecular contact with the surface of the material to be bonded [13], which it can fully achieve if it can wet the surface well, and there will be no case where the adhesive and the bonded material are separated by more than five angstroms. The surface tension of the adhesive is less than the surface tension of the surface to be bonded [14]. This is another potential problem with carbon build-up since if there is oil on the surface of the material to be bonded, the adhesive will tend to spread over the surface of the oil since the surface tension of the oil is more likely to be higher than that of the material to be bonded [15]. In our research, we want to increase this surface tension by surface treatment.

Another theory is the mechanical theory [16], according to which the surface to be bonded is never completely flat, so there are many peaks and valleys on the surface [17]. At one time, it was pseudo-suggested that adhesion was only created by the adhesive flowing into these voids, filling the microcavities, and when it made contact, the adhesive mechanically held the two bonded surfaces together. So, according to mechanical theory, bonding will be at its best when the adhesive can flow into the imperfections, squeeze trapped air out of the interfaces, and mechanically “grip” onto these imperfections. One thing that surface roughness does help adhesion is that it allows the adhesive to encounter a relatively larger area of the material to be bonded [18]. This aids adhesive bonding because, if adhesion is the basis of adhesion—which is based on intermolecular, or interfacial, attractive forces—then if you increase the area of the surface of the adhesive and the material to be bonded where they come into contact, then this increases the area where adhesion forces can develop. Mechanical theory also suggests that the better bonding arrangement is one where the larger the area of contact between the surfaces to be bonded, the greater the area of contact. It depends on the workpiece temperature where the adhesion will be created and its measurement method [19]. In addition, the rougher the interface, the more the roughness can act as a wedge [20] so that the bond can better resist forces that would try to shear the bond [21].

In our research, we will examine the surface roughness of the treated sample after surface treatment and investigate the effect it had on the change in bonding technology. The following theory is the electrostatic and diffusion theory [22]. This theory is not as highly regarded as the mechanical or adsorption theory [23], but there are some cases where it is the most appropriate [24]. The electrostatic theory states that electrostatic forces are formed as an electrostatic electric double layer at the interfaces of the adhesive and the bonded material. This theory is supported by the observation of electrical discharges when adhesive is released from a surface. The electrostatic adhesion theory is accepted in the study of the adhesion of biological cells.

The basic idea of diffusion theory is that adhesion is formed by diffusion between the adhesive and the molecules of the bonded surface. This theory is primarily applicable when both the adhesive and the bonded surface are polymers, which have long chains of molecules that can move. The key is that the molecules of the adhesive and the bonded material must be chemically compatible with each other in terms of diffusion and intermixing.

The last theory of adhesion is the weak interface theory [25]. This theory was first described by Bikerman, who argued that when the bond appears to have failed at the interface, it is usually a weak interface layer cohesive failure in reality [26]. This theory states that bond failure due to interface failure is actually very rare. The failure is so close to the interface that it appears to be the interface that fails, but in most cases, it is the deformation or cohesive failure of a weak interface layer of flexible plastic. These weak interface layers can be created by the adhesive, the bonded material, the environment, or a combination of the three. There are three phases of adhesive bonding: the application of the adhesive, its curing, and the period it is in use. At any of these times, these weak interfaces can form.

Weak interface interfaces can be removed by surface treatment methods, and surface treatment methods can improve the surface energy of the substrate.

Table 1 shows the maximum achieved tensile strength of structural adhesives in the past and in the present.

High-strength adhesives have evolved enormously over the past two decades, thanks to the huge development of the industry and the widespread use of adhesives as a bonding technology. This development is illustrated in Table 1, which shows that adhesive manufacturers have managed to develop the strongest bonded joint seven times in the last 12 years. In 2007, the industry was using adhesives in a small number of structures, so the strength of adhesive bonds was well below later versions, with the maximum load capacity of high-strength adhesives being 9.6 MPa. The year 2009 saw the start of a development that is continuing today. This year, the development limit was broken twice, and by the end of the year, the maximum load strength of bonded joints was 12.4 MPa. The 2010s saw a major development in the industry, which led to a surge in demand for structural adhesives, requiring further advances in high-strength bonding technology. For 2012, the maximum tensile strength of bonded joints increased by almost 10.4 MPa compared to the 2009 result. By that time, the strongest adhesive had 24.7 MPa tensile strength on the surface. A year later, in 2013, the figure more than doubled from the 40.2 MPa achieved in 2011 to 20 MPa. At that time, no distributor could beat this figure for years. However, in 2019, a German company that manufactures and distributes adhesives developed an adhesive with 43.1 MPa tensile strength. In just 12 years, the tensile strength limit of epoxy adhesives has been more than quadrupled [27].

In their research, K. Leena and her team investigated the bonding strength of aluminum alloy under various surface treatments. They used B51SWP aluminum alloy and epoxy resin adhesive for the study. Initially, each specimen was sanded with P100 sandpaper, followed by cleaning to remove residues and contaminants. Three different surface treatments were applied: the first involved degreasing with a solvent (trichloroethylene solution) and air-drying below 60 °C. The second treatment immersed the specimens in a 60 °C acidic solution for 15 min, followed by rinsing with tap water and air-drying. The third treatment involved immersion in BR127 primer at room temperature for 30 min, followed by 30 min of drying in a 120 °C oven. Surface energy, surface topography, wetting contact angle, and bond strength were examined. The primer treatment yielded the best results in terms of dispersive surface energy. Regarding total surface energy and polar component, the acid-treated surfaces showed the best outcomes (mN/m). The primer-treated surfaces exhibited the most favorable wetting contact angle (25°), while the first method resulted in the highest (least favorable) wetting contact angle (65°). Chemically treated and bonded specimens showed the highest tensile strength (19 MPa), with primer-treated surfaces having a tensile strength of 18 MPa. However, the adhesion work was best for the primer-treated surface [28]. At the National School of Engineering in Monastir, Tunisia, Yasmina Boutar and her research group examined the effects of various surface treatments on aluminum adhesive bonds. They used aluminum–copper alloy and one-component polyurethane adhesive in accordance with ASTM D1002 standards [29]. The surfaces were degreased with acetone-based cleaner and treated with a primer, followed by 15 min of drying. Shear strength tests were performed on the bonded specimens. Results showed that the specimens with a 0.6 μm surface roughness exhibited the highest average shear strength of 3.97 MPa. The 0.6 μm surface roughness specimens also showed the most favorable wetting contact angle of 62.01° [30]. Sergio Correia and his research team investigated the effects of chemical surface treatments on the strength of bonded joints. They applied phosphoric acid anodization, chromic acid anodization, sulfuric acid anodization, and boron-sulfuric acid anodization. After chemical treatments, surfaces were primed, and bond strength was assessed. Specimens treated with sulfuric acid and bonded with AF 163-2 adhesive yielded the highest bond strength of 25 MPa [31]. A. Spaggiari and his team examined the strength of aluminum bonded joints under three different surface treatment methods. Surface treatments included manual sanding, sandblasting with fine alumina particles, and diamond-tipped milling. After treatments, Loctite 7063 was used to remove surface contaminants, and immediate bonding was required to prevent oxidation. Single-lap and double-lap joints were tested, with results showing the highest shear strength of 15 MPa for single-lap joints with a sanded surface [32]. Chi-Vinh Ngo and Doo-Man Chum in South Korea investigated changes in aluminum surface energy under various mechanical surface treatments. They applied laser treatment, heat treatment, and hot water treatment to aluminum surfaces. Laser treatments resulted in a consistent lattice pattern, and subsequent heat or hot water treatments had minimal effect on the nanoscale structure. Laser-treated aluminum showed a contact angle of 42 ± 1°, which increased after heat treatment. Laser-treated aluminum surfaces exhibited superhydrophobic properties after 120 min of hot water treatment, demonstrating stability even after 2 months. The study also examined water droplet spreading time, showing improved superhydrophilic performance with longer hot water treatment [33]. Rodríguez-Villanueva and team employed plasma cleaning on traditional steel, intentionally contaminating the surface with oil. Plasma treatment increased the shear strength of silicone-based adhesive bonds from 1.9 MPa to 2.3 MPa. However, plasma treatment on metals was found to be less effective compared to polymers [34]. Tang and colleagues treated AISI 304L stainless steel with atmospheric pressure plasma for various durations. Surface energy increased over time, reaching a maximum of 72 mN/m with a 9° wetting contact angle. Plasma treatment duration impacted the effectiveness, suggesting that in some cases, plasma surface activation might require a longer duration [35].

The objective of our research is to examine the adhesion of high-strength epoxy adhesive on different surface treatment states of steel material. We investigated three distinct surface conditions, namely, control, sandblasted, and surface-activated by cold plasma. Our focus was to explore ways to further enhance the strength compared to the control surface. Additionally, we analyzed the effects of surface roughness, surface wettability, activation level, microtopography, and changes in chemical composition as determined by elemental analysis on the adhesive’s surface adhesion. In this study, we aim to investigate the possibilities of increasing the tensile strength of bonded joints through an understanding of these relationships. The findings presented in this scientific article contribute to the broader understanding of enhancing adhesion strength in epoxy-bonded joints.

## 2. Materials and Methods

### 2.1. Steel Rod for the Specimen

In our experimental setup, steel rods constituted the subject of examination, scrutinized through the Oxford Instruments (Oxford, UK,) Foundry Master Pro optical emission spectrometer. Prior to conducting the research, the spectrometer underwent meticulous calibration to ensure optimal performance and adequate resolution. The spectrometer can measure values of the selected chemical elements precisely within the range of 0.01–0.0001. Consequently, the instrument was adept at providing accurate readings, a crucial attribute facilitated by the calibration processes. The ensuing results, expressed in weight percentage (wt%), are presented in Table 2.

From the spectroscopy of the steel bar, it can be concluded that the steel we have tested matches the material composition of 90MnCrV8 (1.2842) cold-worked tool steel. This type of steel, besides its iron content of 96.1%, contains additional alloying elements: 0.85–0.95% carbon, 0.1–0.4% silicon, 1.8–2.2% manganese, 0.15–0.5% chromium, and 0.05–0.2% vanadium. In terms of its uses, it is preferably used as a raw material for cutting and punching tools (for medium plate thicknesses), deep drawing tools, threading and reaming tools, measuring instruments, milling tools, and their ejector pins. The hardness of the material is close to 65 HRC, which can be further increased by appropriate heat treatment (heat treatment 780–800 °C, cooling in oil, tempering 170–190 °C). Thanks to its wide range of applications, the material is available in flat steel, steel bar, and plate.

### 2.2. Cold Plasma

For the surface treatment, we used PlasmaTreat GmbH Germany OpenAir^®^ FG5001 (Steinhagen, Germany) atmospheric pressure compressed air system. The cold plasma was generated by a plasma generator with a maximum output power of 600 W. A plasma head and a high-voltage transformer were connected to the generator. The machine was operated at 21 kHz. The exit point of the plasma was eccentrically positioned at the edge of the head and rotated at 2500 rpm to dissipate the energy uniformly in the scattering radius. The compressed air flow was set to 30 L/min. Previous research results show that using steel also can be treated but with lower effectiveness. Process settings were the distance between the plane of the material to be treated and the plasma-emitting head and the speed of the main straight-line motion with a value of 5 mm distance and 0.5 mm/s movement speed.

### 2.3. Surface Treatments

Before carrying out the surface modification processes, the steel bar is cut with a bandsaw for the tensile test specimens, sized to the specific design to the required dimensions. We cleaned the cut pieces with alcohol to remove any dirt from cutting, storage, and preparation. For this paper, we investigated 3 different surface treatment and preparation methods labeled A, B, and C.

#### 2.3.1. Turned and Alcohol-Cleaned Surface—Type A

For turned surfaces, steel bars were cut to size and then cleaned with alcohol by means of an E400 universal lathe at 750 rpm while the correct amount of coolant and the optimum feed rate to the predetermined dimensions were used. It was followed by another cleaning process.

#### 2.3.2. Turned, Sandblasted, and Alcohol-Cleaned Surface—Type B

Before sandblasting, the samples were prepared as summarized in the previous paragraph (Section 2.3.1). After cleaning, sandblasting was carried out as an additional surface treatment. During the treatment, the grains were impacted against the surface at an angle of about 45°. The sandblasting was carried out with quartz particles of 300 μm. After the treatment, the surface was cleaned with alcohol to remove the deposited dust and sand particles and then dried with a drying machine.

#### 2.3.3. Turned, Sandblasted, Alcohol-Cleaned, and Plasma-Treated Surface—Type C

The turned, sandblasted, alcohol-cleaned, and plasma-treated specimens were prepared before the plasma treatment, as summarized in Section 2.3.1 and Section 2.3.2. The distance between the end of the nozzle and the surface to be treated was 5 mm during the plasma treatment. During the treatment, the nozzle rotating speed was set to 2500 rpm, the air flow rate to 30 L/min, and the treatment power to 405 W. The feed rate of the plasma source and, with it, the plasma jet was controlled by a robot at 0.5 mm/second. No further cleaning process was applied after the treatment.

### 2.4. High-Strength Adhesive

For the adhesive joints tested in this paper, we used Henkel Hungary Ltd. Loctite^®^ EA9514 adhesive (Budapest, Hungary). Henkel Hungary Ltd. Loctite^®^ EA9514 is a hardened, one-component, thermosetting epoxy adhesive, which is especially suited for industrial applications such as bonding filter units, magnets, structural components, and is also well suited for high gap filling and high operating temperature applications. The advantages of Henkel Hungary Ltd. Loctite^®^ EA9514 adhesive include suitable adhesion on a variety of surfaces, optimum gap-filling ability, high peel and tensile strength, increased toughness, and excellent heat and chemical resistance. The adhesive is suitable for bonding different types of steel, aluminum, and copper surfaces. The adhesive crosslinks under the influence of air temperature. The manufacturer’s recommended curing temperature is between 120 °C and 175 °C, which should be maintained for approximately 30–60 min for proper curing. In terms of its application environment, it has a high resistance to room-temperature water, salt water, acids, alkalis, and oils. The substrate must be clean, dry, and free from grease, oil, dust, and dirt before bonding. Henkel Hungary Ltd. Loctite^®^ EA9514 can be applied to the surface using manual, compressed air, electric spray guns, and a pump system. According to the product data sheet provided by the manufacturer, the adhesive has a tensile strength of 44 MPa, a compressive strength of up to 66 MPa, and an elongation at break of 5.8%. Furthermore, it has a thermal conductivity of 0.3 W/(m∙K). The recommended use temperature is 2–8 °C, and the application temperature −30–200 °C. At extreme temperatures, the adhesive is resistant for nearly 1500 h (150 °C), and at higher temperatures (200 °C), it reaches 50% of its strength after a continuous reduction for 3000 h.

### 2.5. Jig for the Adhesive Joint

**Figure 1:**
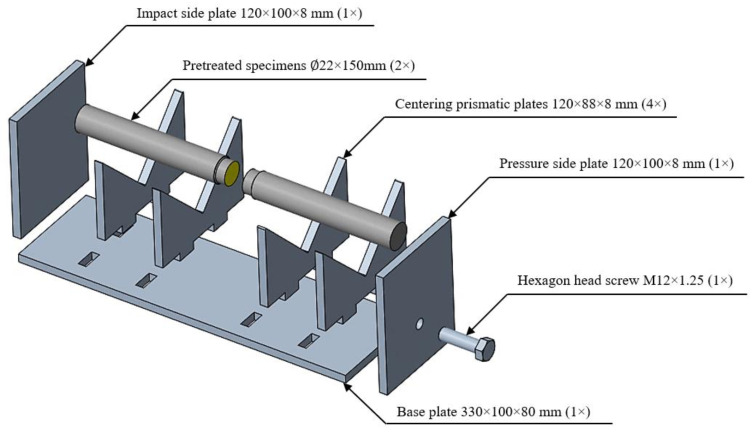
Jig for the adhesive joint.

A centering device has been designed in order to ensure the repeatability of the specimens prepared for tensile strength tests, to glue them in one plane, to stabilize the clamping force, to tighten torque, and to reduce the variance of the results. The basic requirements established included proper centering of the test specimens, the existence of a constant, repeatable bond gap, tightening torque, and the ability to apply the device to subsequent changes in the diameter of the lap steel after a simple drilling and tapping process. To ensure optimal gripping and neutralization of all geometric errors, meticulous measures were taken in the operation of the device. Every aspect of the apparatus was systematically addressed to achieve precise and secure clamping. Rigorous attention was given to neutralizing any inherent geometric discrepancies, acknowledging the critical role they play in the overall performance and accuracy of the equipment. This comprehensive approach not only guarantees an effective grip but also underscores the commitment to mitigating potential sources of error, affirming the reliability and precision of the device in its intended applications. Other requirements included the heat resistance of the device and its resistance to form during heat treatment processes. As a first step of the design task, the various elements of the device were designed and dimensioned in PTC Creo 7 C3D V 2.5 CAD software. After editing, a band plan was created, which allowed us to work with the highest material removal factor during laser cutting. During the cutting process, thanks to the setting of the right parameters, we obtained an almost mirror surface along the cut contours, thus ensuring the accuracy of the device and the best possible centering. The impact side plate and the pressure side plate were fixed to the base plate by means of welded joints. During welding, care was taken to maintain the rigidity of the plates. The centering prismatic plates were then fitted into their positions on the base plate. Finally, a hexagon head screw (M12 × 1.25) was driven into the threaded hole in the pressure side plate. Thanks to the centering device, we were able to produce accurate, aligned specimens with a suitable bonding gap (Figure 1).

### 2.6. Adhesive Technology

**Figure 2:**
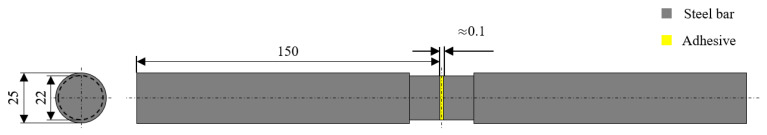
Geometry of tensile test specimen and adhesive.

During the research, the specimens for the tensile strength tests were prepared by developing unique parameters and dimensions. Consequently, the diameter of the bonded area was 22 mm. The maximum diameter of the specimens was 25 mm, and their length was 150 mm each (Figure 2).

To carry out the tests, we clamped the workpiece in the lower clamping head of the testing machine using the cylindrical clamping jaws, then, using the specimen protection (<50 N force), we clamped the workpiece in the crosshead as well, and then, holding the clamped circular steel, we carried out the tensile strength tests of the bonded joints. To ensure the repeatability of the 0.1 mm bonding gap and to maintain the bonding area, we used a centering device, where the steel bars to be bonded were aligned in a plane thanks to the prismatic plates, and we ensured a constant bonding gap by tightening the screw provided with the device to a torque of 2 Nm. During the whole time of the adhesive curing, i.e., the entire heat treatment, the test pieces were tightened to the specified torque in the apparatus. A pulling speed of 2 mm/min was used for the tensile strength tests.

### 2.7. Tensile Test of the Adhesive

**Figure 3:**
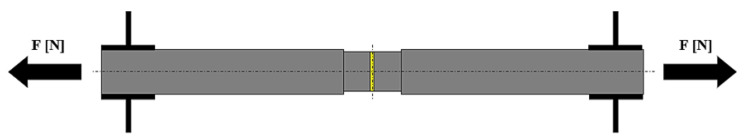
Illustration of tensile test of high-strength adhesive on steel bars.

The bonded joints were tensile tested using an INSTRON 5900R 4482 (Norwood, MA, USA) universal testing machine. The tensile speed was 2 mm/min. To avoid side pulling, we used a cylindrical gripper head during the clamping. The tensile strength of the bonded joints of the specimens was characterized by the average stress at the bonded surface. Seven specimens were used for each experimented parameter. Figure 3 shows the arrangement of the tests.

### 2.8. Surface Roughness Measurement

The surface roughness values altered by the surface treatments were also investigated using the Olympus LEXT OLS5000 (Westborough, MA, USA) confocal laser scanning microscope. The measuring equipment allows 3D surface imaging, a highly efficient method for detailed analysis of microstructures and surfaces. The measurements are performed by a diode laser source operating at a wavelength of 405 nm. The measurement accuracy is ±5% + 1 nm in the vertical direction, which is extremely accurate and close to the true roughness of the surface. Furthermore, it has three types of measurement and imaging speeds: fast (10 frames/s), normal (2.5 frames/s), and high resolution (slow) (0.5 frames/s). A magnification of 20× was used during the measurements to obtain images of sufficient quality to obtain results closest to the true values. The profile method type measurement was performed according to ISO 21920-2:2021 [36] and the area type ISO 25178-2 [37]. The tests were repeated three times and carried out in different areas on the surfaces.

### 2.9. Surface Wetting Measurement

Immediately after the surface treatments, we measured the wetting contact angle on the specimens at room temperature using the stilling-drop method. Using a micropipette, 5 µL drops were used for the measurement. Two liquids were used for the droplet analysis to apply the Fowkes method: distilled water and ≥99% pure ethylene glycol.

### 2.10. SEM Investigation

For the SEM investigations, a ZEISS Sigma 300 (Cambridge, UK) variable pressure microscope was used. We analyzed the surface of the specimens with in-lens SE, BSD, and EDX detectors. For the SE and BSD cases, a 10 kV accelerating voltage was used. From each sample, different magnifications were used from 100× to 50,000×.

### 2.11. Heat Curing

A custom-built laboratory heat treatment furnace was used with a HAGA KD481P (Budapest, Hungary) control unit with hot air mixing; additionally, we checked the temperature with a thermometer near the bonding. The examined curing parameters were 90 min and 165 °C, 30 min and 175 °C, and 45 min and 190 °C.

## 3. Results

### 3.1. Effect of Heat Curing Duration and Temperature on Strength

**Figure 4:**
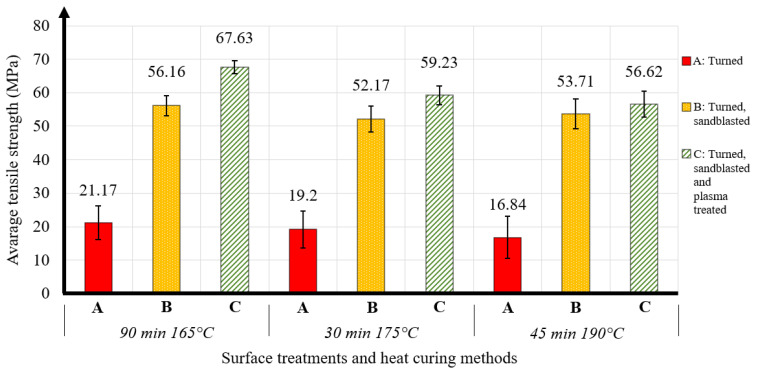
Effect of heat curing and temperature of using high-strength adhesive on steel.

In our research, we have also tested the change in strength of bonded joints with different heat treatment methods on specimens that have been surface treated in different ways. Figure 4 illustrates the effect of different surface treatment and heat treatment methods as a function of the average tensile strength values obtained.

It can be clearly observed that the tensile strength of the bonded joints decreased with increasing curing temperature. The most unfavorable values were obtained with specimens annealed at 45 min and 190 °C. Heat treatment at 30 min and 175 °C resulted in an improvement in strength values. Similarly, the most favorable average tensile strength values were obtained after 90 min and 165 °C heat treatment. Furthermore, it can be noticed that type C, i.e., turned, sandblasted, and plasma-treated surfaces, provided the highest tensile strength, whereas the specimens made of turned surfaces had the lowest strength. As the highest tensile strength values were obtained after 90 min and 165 °C heat treatment, we will now try to achieve the maximum strength values with this type of specimen.

### 3.2. Average Strengths Achieved with Optimal Surface Treatment and Heat Curing Technology

**Figure 5:**
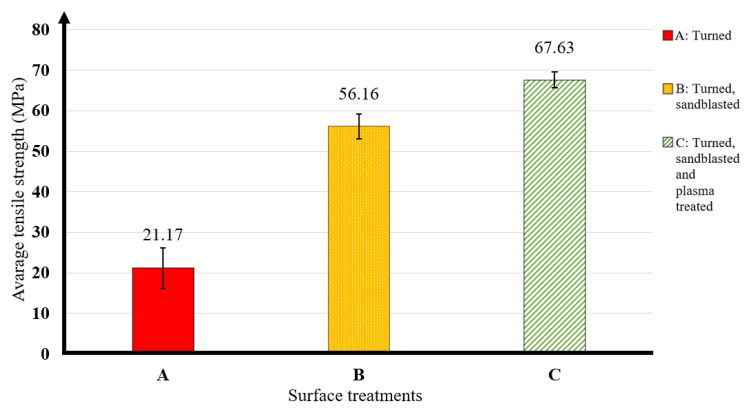
Effect of surface treatments on tensile strength of adhesive joined steels.

As shown in the previous graph, the average tensile strength results were obtained after 90 min and 165 °C heat curing. Figure 5 and Table 3 illustrate the average tensile strength results obtained for specimens treated with this type of heat treatment as a function of the different surface treatments.

The bonded test specimen made from the turned surface achieved an average load of 21.17 MPa. For the turned and sandblasted surface, an average load capacity of 56.16 MPa was obtained. For the turned, sandblasted, and plasma-treated surface, an average tensile strength of 67.63 MPa was obtained. The most outstanding result of our research was the tensile strength of this bonded joint.

#### Results of Load-Holding Tests

Table 4 shows that we examined the holding time of the joints while the load value was fixed. For this test, we used the C type, which produced the most tensile strength from the specimens. It is visible that if we lower the strength of the load, then we could achieve a significantly higher holding time.

## 4. Discussion

In this chapter, the reasons for the results showing the highest tensile strength are extracted through surface topography, roughness characteristics, surface wetting, and chemical elemental analysis.

### 4.1. Result of Surface Roughness Measurements

The surface roughness values of specimens’ surfaces treated with different technologies were determined by 3D laser surface imaging. The line scans were measured at 3 mm in length. The measurement values can be seen in Table 5.

**Figure 6:**
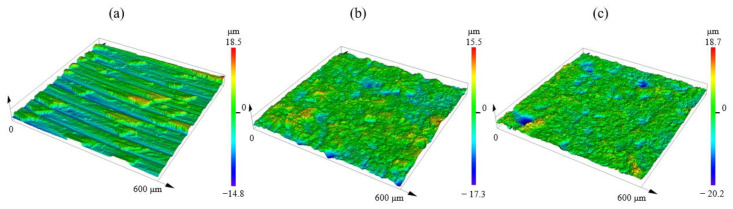
Three-dimensional laser-scanned surface microtopography of adhesion surface on steel bar with different surface treatments according to area method ISO 25178-2: (**a**): turned; (**b**): turned and sandblasted; (**c**) turned, sandblasted, and cold plasma-treated surface.

In terms of the average surface roughness, the turned (A) type surface yielded the highest surface roughness value of 3 μm. For the turned and sandblasted (B) surface, the surface roughness value decreased to 2.2 μm due to sandblasting. Furthermore, the surface treated with lathed, sandblasted, and plasma sprayed (C) showed a decrease in roughness of 0.1 μm compared to the surface marked B. There are two different explanations for this slight decrease. Firstly, presumably, the plasma jet treatment removed the particles and grains left on the surface from the sandblasting, which remained on the surface after cleaning. The second hypothesis is that the roughness of the surfaces treated by the different processes was measured on separate samples. The investigation was conducted on a microtopography scale using 600 × 600 μm^2^ areas. There is a difference between the microtopography of turning and sandblasting operations (Figure 6).

By examining the effect of the change in surface roughness on the tensile strength results, we can conclude that they are indeed closely related. For specimens made from the control (turned) surface, a strength of 21.17 MPa and a surface roughness of 3 μm were measured. Sandblasting reduced the surface roughness and increased the bond strength to 56.16 MPa. The increased strength is presumably also because the sandblasting homogenized the surface, created a finer structural microstructure, and increased the relative surface area. As a result, the adhesive was able to better adhere to the surface microstructure. The plasma surface treatment did not change the roughness of the surface compared to the surface marked B but further increased the strength of the bonded joint (67.63 MPa). The improvement in tensile strength values may also be due to changes in the wettability and carbon/oxygen (C/O) ratio of the surfaces because of the surface treatments. The effect of these on the strength will be investigated further.

### 4.2. Wetting, Surface Energy Measurements

Following the surface roughness test, the wettability and surface energy of the treated surfaces were measured. For the turned surface, i.e., the control surface, a contact angle of 73.1° was measured with distilled water and 51.3° (Table 6) with ethylene glycol, representing a surface energy of 31.9 mN/m. For the turned and sandblasted surface, the magnitude of the contact angles was 47.6° with distilled water and 33.9° with ethylene glycol. This resulted in a total surface energy of 53.3 mN/m (Table 7). This also demonstrates that the sand spraying homogenized the surface and increased the relative surface area, allowing the droplets to spread over the surface, which resulted in a decrease in the contact angle values compared to the control surface. The combined effect of a more homogeneous surface roughness, which was more optimal for bonding, and improved wettability resulted in a large increase in the tensile strength of the bond. On the turned, sandblasted, and plasma-treated surface (C), we obtained extremely suitable values of the contact angle of 7.4° (distilled water) and 0° (ethylene glycol). The surface energy of 86.6 mN/m is close to a perfect hydrophilic surface. In our opinion, the reason for this is that the homogeneous microstructure environment produced by sand spraying was even subjected to a plasma surface activation with a tested parameter. As no surface roughness change was induced by the plasma treatment, the increase in strength of 11.47 MPa was only due to the surface activation effect of the plasma treatment. The change in the ratio of C/O to Fe/O on the surface due to the plasma treatment will be investigated further.

### 4.3. SEM Measurements: SE, BSD, EDX

**Figure 7:**
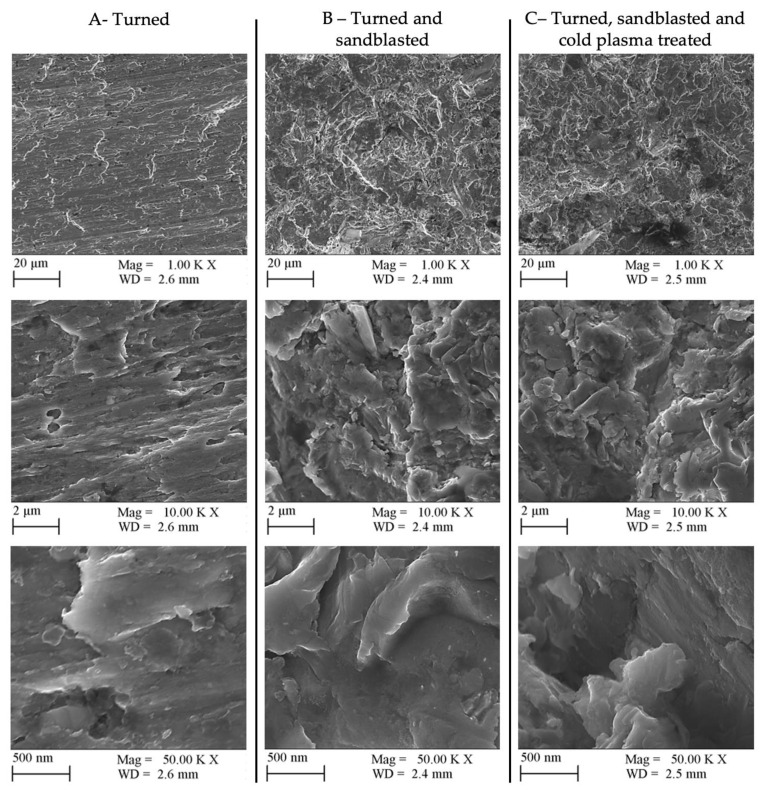
In-lens SE SEM images of different surface-treated samples with 1000×, 10,000×, and 50,000× magnification.

The surface images formed with the secondary electrons of the electron microscope are shown in Figure 7.

The surface structure formed during the turning process is clearly visible on the turned surface, but no other factor affecting the results was detected at any magnification. The images of the turned and sandblasted surfaces show the homogeneous surface created by sandblasting but no evidence of the circular structure created by turning. On the other hand, it is important to note that, in our opinion, the 50.00 K× magnification image reveals the presence of nanoscale silica impurities deposited on the surface during sandblasting, which remained on the surface after cleaning. These probable impurities were not detected in the macro (10.00 K×) image but are already detectable in the micro (50.00 K×) image. In the case of surfaces that underwent turning, sandblasting, and plasma treatment, there is no discernible alteration in the surface structure following the application of the plasma treatment. However, there was a noticeable reduction in the number of nanostructured impurities on the surface, as confirmed by elemental analysis (Table 8). This surface impurities reduction was presumably due to the dynamic cleaning effect of the cold plasma jet treatment.

It is believed that the surface roughness difference of 0.1 μm between surfaces B and C was due to the silica particles on the surface. Furthermore, it is possible that the grain impurities also had an influence on the tensile strength values of the bonded joints.

A ratio of 44.55% carbon and 55.45% oxygen was found on the turned surface (Table 9).

Compared to the control surface, a difference of 0.9% is observed for the sandblasted surface. Since the sandblasting did not change the chemical elemental composition of the surface, the very small percentage difference could be a measurement variance. On the other hand, after the plasma treatment, a greatly reduced carbon percentage (24.85%) and an outstanding oxygen composition of 75.15% were measured. In our opinion, the surface molecular groups were changed by the plasma treatment, and carbon–carbon and carbon–hydrogen bonds (carboxyl groups) were replaced by carbon–oxygen molecular groups. It is probable that due to the high oxygen evolution and the homogeneous increased relative surface area, a near hydrophilic, high surface energy value was measured, and an extremely high tensile strength value of 67.63 MPa was obtained.

The iron/oxygen ratio was first measured on the turned (control) surface, which yielded a weight ratio of 97.6% iron and 2.4% oxygen (Table 10).

In comparison, after sandblasting, the iron content decreased by 10.45%, and the oxygen content increased. In this case, the chemical elemental composition of the surface did not change either, so the change in the ratio could be due to an increase in the relative surface area. Presumably, the homogeneous surface microstructure created by the sand spraying, which has a larger relative surface area than the turned surface, allows more oxygen/oxide to be formed. For the C-type surface, a ratio change of 0.95% was observed. Presumably, the decrease in the weight percent of iron and the increase in the weight percent of oxygen observed for the sandblasted and plasma-coated surfaces can be explained by the increase in the relative surface area. The increase in oxygen percentage and relative surface area also improved the wettability of the surface, as the droplet was able to spread better over the surface. Furthermore, the plasma treatment activated the surface, as evidenced by the large change in the C/O ratio, and the presence of this large amount of oxygen and the topography of the surface allowed us to maximize the strength to 67.63 MPa.

In comparing our research findings with the existing literature, notable connections and correlations emerge, shedding light on the intricate dynamics influencing the strength of adhesive joints in steel applications. The literature emphasizes various surface treatments for aluminum alloys, with studies by Leena et al. [28], Boutar et al. [30], Correia et al. [31], Spaggiari et al. [32], Ngo and Chum [33], and Rodríguez-Villanueva et al. [34] employing techniques such as degreasing, acid treatments, primer application, sanding, and plasma cleaning. Our results echo the significance of surface treatment, particularly showcasing the pivotal role of turned, sandblasted, and plasma-treated surfaces (C) in achieving optimal tensile strength. The literature underscores the impact of surface energy and wetting contact angle on bond strength, aligning with our observations of enhanced wettability and substantial strength in the C-type surface. The correlation between surface roughness and adhesive strength is highlighted in both the literature and our research. Furthermore, our investigation into the effects of heat curing aligns with the broader literature, emphasizing the crucial role of curing temperature and duration on the mechanical properties of bonded joints. Noteworthy is the consistency in optimal conditions across studies, with both the literature and our research pointing toward improved strength at specific heat-curing parameters. The nuanced exploration of chemical composition, exemplified by the C/O and Fe/O ratios in our study, resonates with the literature’s emphasis on the impact of chemical treatments on bond strength. The comprehensive understanding achieved in our research aligns with the literature, collectively contributing insights into surface engineering for adhesive applications in steel joints. This interconnected body of knowledge enhances the practical implications for optimizing adhesive performance in steel applications, underscoring the significance of surface treatments, wetting characteristics, and chemical composition in achieving superior tensile strength. The current literature provides limited information on tensile strength connections, particularly in cases where pull is the primary load. Our findings offer valuable advancements in adhesive technology during tensile load state.

The results of our study reveal a nuanced and interconnected relationship between surface topography, wetting properties, chemical composition, and tensile strength in adhesive joints. The observed intricacies underscore the following conclusions: Surface roughness is a pivotal determinant of adhesive strength. Increased roughness, as seen in turned surfaces, corresponds to lower tensile strength. Sandblasting significantly reduces roughness, contributing to improved adhesive strength. Wetting characteristics transition from hydrophobic to hydrophilic behavior. Enhanced wetting, exemplified in the turned, sandblasted, and plasma-treated surface (C), is associated with improved adhesive strength. The homogeneity induced by sandblasting and subsequent plasma treatment contributes to superior wetting. Alterations in the C/O and Fe/O ratios induced by plasma treatment correlate with changes in surface chemistry. The turned, sandblasted, and plasma-treated surface (C) exhibits a unique C/O ratio and exceptional tensile strength, emphasizing the impact of chemical composition on adhesive strength. Increased oxygen percentage and modifications in chemical groups contribute to improved wettability and tensile strength. The turned, sandblasted, and plasma-treated surface (C) consistently demonstrates the highest tensile strength. Optimal conditions for tensile strength are achieved at 90 min and 165 °C, showcasing the influence of heat treatment on adhesive performance. In synthesis, our findings highlight the multifaceted interplay between surface topography, wetting behavior, chemical composition, and tensile strength in adhesive joints. The turned, sandblasted, and plasma-treated surface emerges as a configuration that optimizes these interconnected factors, providing valuable insights for the design and enhancement of adhesive systems in steel applications. This comprehensive understanding contributes to the broader knowledge of surface engineering and materials science, offering practical implications for adhesive performance optimization. The new findings of this work are in its holistic exploration of the complex interrelationships among surface topography, wetting properties, chemical composition, and tensile strength in adhesive joints. The study uniquely identifies the turned, sandblasted, and plasma-treated surface (C) as an optimal configuration for maximizing tensile strength, providing practical implications for adhesive applications in steel joints. The findings reveal interconnected aspects, showcasing how changes in surface roughness and chemical composition influence multiple factors simultaneously, contributing a layer of complexity to the existing knowledge. This comprehensive and application-oriented approach adds practical value and advances the understanding of adhesive behavior in steel joints, establishing the distinctiveness and significance of the research.

## 5. Conclusions

Our systematic investigation into the impact of diverse heat treatment methodologies and surface treatments on the tensile strength of adhesive joints in steel has culminated in a series of quantifiable outcomes. The discerned findings, characterized by precise numerical values, elucidate the following critical facets:

- Evident is a discernible reduction in tensile strength with escalating annealing temperatures. The pinnacle performance is achieved at 90 min and 165 °C, manifesting a superlative tensile strength of 67.63 MPa. In stark contrast, specimens annealed at 45 min and 190 °C exhibit a considerably diminished tensile strength of 56.62 MPa;
- Type C surfaces (turned, sandblasted, and plasma treated) consistently manifest superior tensile strength characteristics. The turned surface epitomizes the nadir of strength at 21.17 MPa, whereas the turned and sandblasted surface attains an elevated strength of 56.16 MPa. The apogee is reached by the turned, sandblasted, and plasma-treated surface (C), registering an exemplary tensile strength of 67.63 MPa;
- Load-holding tests conducted on the turned, sandblasted, and plasma-treated surface (C) unveil commendable endurance under varied load conditions, recording strengths of 49 MPa for 38 min and 47 MPa for 65 min;
- Surface roughness analysis delineates the turned surface as possessing the maximal roughness (Ra = 3 μm), whereas sandblasting engenders a reduction to 2.2 μm. The turned, sandblasted, and plasma-treated surface (C) manifests a marginal decrease in roughness to 2.1 μm;
- Wetting and surface energy assessments showcase near-perfect hydrophilicity for the turned, sandblasted, and plasma-treated surface (C), manifested by contact angles of 7.4° (distilled water) and 0° (ethylene glycol), coupled with a surface energy of 86.6 mN/m;
- Scanning electron microscopy (SEM) images validate the homogeneity induced by sandblasting, while energy-dispersive X-ray (EDX) analysis corroborates the cleansing effect of plasma treatment, notably reducing nanostructured impurities on the surface;
- Plasma treatment profoundly alters the C/O and Fe/O ratios, underscoring shifts in surface chemistry. The turned, sandblasted, and plasma-treated surface (C) exemplifies a C/O ratio of 24.85, concomitant with an exceptional tensile strength of 67.63 MPa.

## Figures and Tables

**Table 1:** Previously achieved tensile strength of bonded joints using high-strength adhesive [27].

Year	Development of Tensile Strength of Bonded Joints (MPa)
2007	9.6
2009	12.4
2009	15.2
2011	20
2012	24.7
2013	40.2
2019	43.1

**Table 2:** Material composition of the steel rod.

Fe	C	Si	Mn	P	S	Cr	Mo	Ni
96.1	0.937	0.15	2.07	<0.0009	0.0042	0.161	0.0118	0.0405
Al	Co	Cu	Nb	Ti	V	W	Pb	Sn
0.0172	0.0075	0.112	0.0178	0.0029	0.182	<0.008	0.0029	0.0126
B	Ca	Zr	Bi	As	N	Sb		
0.0041	0.0006	0.0036	<0.005	0.0066	0.0607	<0.003		

**Table 3:** Average tensile strength values of adhesive joined surface-treated steel.

Surface Treatments	Marking	Average Tensile Strength (MPa)
Turned surface	A	21.17 ± 5
Turned and sandblasted surface	B	56.16 ± 3
Turned, sandblasted, and plasma-treated surface	C	67.63 ± 2

**Table 4:** Results of the load-holding tests on the highest adhesion surface.

Surface Treatment	Marking	Strength Load (MPa)	Time (min)
Turned, sandblasted, and plasma-treated surface	C	49 47	38 65

**Table 5:** Surface roughness values of the different surface-treated samples according to ISO 4287 profile measurement method.

Surface Treatments	Label	R_a_ (μm)	R_z_ (μm)
Turned surface	A	3	15.7
Turned and sandblasted surface	B	2.2	13
Turned, sandblasted, and plasma-treated surface	C	2.1	14.8

**Table 6:** Result values of surface wettability measurement.

Surface Treatments	Marking	Contact Angle Distilled Water (°)	Contact Angle Ethylene Glycol (°)
Turned surface	A	73.1	51.3
Turned and sandblasted surface	B	47.6	33.9
Turned, sandblasted, and plasma-treated surface	C	7.4	0

**Table 7:** The result values of surface energy measurements on steel.

Surface Treatments	Marking	Surface Disperse Component (mN/m)	Surface Polar Component Value (mN/m)	Total Surface Energy (mN/m)
Turned surface	A	16.5	15.4	31.9
Turned and sandblasted surface	B	7	46.3	53.3
Turned, sandblasted, and plasma-treated surface	C	2.2	84.4	86.6

**Table 8:** EDX measurement results: Fe and Si values using different surface treatments.

Surface Treatments	Marking	Fe/Si Ratio (m/m%) Fe	Fe/Si Ratio (m/m%) Si
Turned and Sandblasted Surface	B	92.4	7.6
Turned, Sandblasted, and Plasma-Treated Surface	C	93.1	6.9

**Table 9:** EDX measurement results: C and O values using different surface treatments.

Surface Treatments	Marking	C/O Ratio (m/m%) C	C/O Ratio (m/m%) O
Turned surface	A	44.55	55.45
Turned and sandblasted surface	B	45.45	54.55
Turned, sandblasted, and plasma-treated surface	C	24.85	75.15

**Table 10:** EDX measurement results: Fe and O values using different surface treatments.

Surface Treatments	Marking	Fe (m/m%)	O (m/m%)
Turned surface	A	97.6	2.4
Turned and sandblasted surface	B	87.15	12.85
Turned, sandblasted, and plasma-treated surface	C	88.1	11.9

## Data Availability

Data are contained within the article.
